# Phenotypic Characterization of Retinoic Acid Differentiated SH-SY5Y Cells by Transcriptional Profiling

**DOI:** 10.1371/journal.pone.0063862

**Published:** 2013-05-28

**Authors:** Joanna A. Korecka, Ronald E. van Kesteren, Eva Blaas, Sonia O. Spitzer, Jorke H. Kamstra, August B. Smit, Dick F. Swaab, Joost Verhaagen, Koen Bossers

**Affiliations:** 1 Department of Neuroregeneration, Netherlands Institute for Neuroscience, An Institute of the Royal Netherlands Academy of Arts and Sciences, Amsterdam, The Netherlands; 2 Department of Molecular and Cellular Neurobiology, Center for Neurogenomics and Cognitive Research, Neuroscience Campus Amsterdam, Vrije Universiteit, Amsterdam, The Netherlands; 3 Institute for Environmental Studies (IVM), Vrije Universiteit, Amsterdam, The Netherlands; 4 Department of Neuropsychiatric Disorders, Netherlands Institute for Neuroscience, An Institute of the Royal Netherlands Academy of Arts and Sciences, Amsterdam, The Netherlands; National University of Singapore, Singapore

## Abstract

Multiple genetic and environmental factors play a role in the development and progression of Parkinson’s disease (PD). The main neuropathological hallmark of PD is the degeneration of dopaminergic (DAergic) neurons in the substantia nigra pars compacta. To study genetic and molecular contributors to the disease process, there is a great need for readily accessible cells with prominent DAergic features that can be used for reproducible *in vitro* cellular screening. Here, we investigated the molecular phenotype of retinoic acid (RA) differentiated SH-SY5Y cells using genome wide transcriptional profiling combined with gene ontology, transcription factor and molecular pathway analysis. We demonstrated that RA induces a general neuronal differentiation program in SH-SY5Y cells and that these cells develop a predominantly mature DAergic-like neurotransmitter phenotype. This phenotype is characterized by increased dopamine levels together with a substantial suppression of other neurotransmitter phenotypes, such as those for noradrenaline, acetylcholine, glutamate, serotonin and histamine. In addition, we show that RA differentiated SH-SY5Y cells express the dopamine and noradrenalin neurotransmitter transporters that are responsible for uptake of MPP(+), a well known DAergic cell toxicant. MPP(+) treatment alters mitochondrial activity according to its proposed cytotoxic effect in DAergic neurons. Taken together, RA differentiated SH-SY5Y cells have a DAergic-like phenotype, and provide a good cellular screening tool to find novel genes or compounds that affect cytotoxic processes that are associated with PD.

## Introduction

Parkinson’s disease (PD) is the second most prevalent age-related neurodegenerative disease. The primary clinical symptoms consist of deficits in motor behavior such as tremor, muscle rigidity, postural instability, akinesia and bradykinesia [1] as well as cognitive dysfunction [2], [3]. The motor symptoms are caused by the selective loss of the dopaminergic (DAergic) neurons in the substantia nigra pars compacta (SN) leading to depletion of striatal dopamine (DA) levels. Several mutations have been found that cause rare, familial forms of PD in genes such as SNCA, PARK2, DJ-1, PINK1, LRRK2 and PARK9 [4], [5]. These familial forms account for only 5% of the patients, whereas most PD cases are sporadic [1]. The etiology of sporadic PD appears to be multifactorial including both genetic and environmental factors [6], [7]. So far, several cellular defects, such as the formation of Lewy bodies [8], [9], mitochondrial dysfunction and increased oxidative stress, have been linked to the disease, although these cannot fully explain the molecular basis of the disease [10].

Recently, several genome-wide gene expression studies on postmortem human brain tissue have identified transcriptional alterations that are associated with sporadic PD [11]–[19]. These alterations may either be causally involved in the development of sporadic PD, or may be the consequence of the progression of the disease. The functional interpretation of the molecular signatures obtained by transcriptional profiling poses a challenge and gene function analysis is dependent on a reliable cellular model enabling large-scale functional screening of genes. An adequate cell model for research on PD gene function should: 1. display the main cellular and molecular features that are characteristic of DAergic neurons, 2. be sensitive to perturbations in cellular processes that are commonly associated with PD, and 3. be suitable for up-scaled cellular screening, in which the function of many genes, proteins and compounds can be examined in a high-throughput and high-content manner.

One potentially suitable cell model is the human neuroblastoma SH-SY5Y cell line, which was originally derived from the SK-N-SH cell line [20]. SH-SY5Y cells have been used frequently, either in an undifferentiated state [21]–[24], or in a neuron-like differentiated state after induction with all-trans-retinoic acid (RA) [25]–[32]. The specific neurotransmitter phenotype of SH-SY5Y cells differentiated with RA is still unclear. RA treatment has been shown to induce the expression of tyrosine hydroxylase (TH), suggesting a shift towards a DA neurotransmitter phenotype [33]. However, others did not observe changes in the expression of key DAergic-cell markers in RA treated cells [21]. RA treatment has also been reported to induce a cholinergic phenotype [34]. The lack of an unequivocal characterization of the transmitter phenotype of RA differentiated SH-SY5Y cells currently impacts on the potential relevance of these cells for PD research.

In 1982, 1-methyl-4-phenyl-1,2,3,6-tetrahydropyridine (MPTP) was discovered as a neurotoxin which induced rapid PD symptoms in exposed humans. Neuropathological examination of the brains of these subjects revealed a moderate to severe loss of DAergic neurons in the SN [35]. In the brains of exposed individuals, 1-methyl-4-phenyl-pyridium (MPP(+)), an active metabolite of MPTP, is taken up by DAergic neurons via the dopamine (DAT) and noradrenaline transporter (NAT) [36], resulting in the inhibition of complex 1 of the mitochondrial electron transport chain, rapid ATP depletion, loss of mitochondrial membrane potential and the formation of reactive oxygen species (ROS) [37], [38], together leading to cellular dysfunction and cell death [39].

MPP(+) treated SH-SY5Y cells have been widely used as an *in vitro* model to study mitochondrial impairments observed in PD. So far, two gene expression profiling studies investigated the effect of MPP(+) treatment on undifferentiated SH-SY5Y cells [22], [23]. In these studies, mitochondrial stress indeed preceded cellular death, but the relevance of these studies for PD research is so far unclear because the neurotransmitter phenotype of these undifferentiated cells was not studied. This is of importance, since both PD and MPP(+) specifically induce cell death in SN DAergic neurons, which suggests an interaction between the DA neurotransmitter phenotype and PD-associated mitochondrial stress. For example, MPP(+) is not only selectively transported into DAergic neurons, but it also binds with high affinity to VMAT2, a protein that transports DA into vesicles. The interaction between VMAT2 and MPP(+) causes excessive release of DA into the cytoplasm, increasing ROS generation, which subsequently contributes to cell death [38]. Thus, it is of importance to know if SH-SY5Y cells display any typical DAergic characteristics that enable the study of MPP(+) toxicity in a PD-like context.

Genome-wide transcriptional analysis of specific neuronal cell types is a powerful method to investigate their molecular properties, ascertain their lineage and study their differentiation characteristics [40], [41]. Here we used large-scale transcriptional profiling combined with gene ontology and pathway analysis to determine the molecular phenotype of SH-SY5Y cells. We then used high-content microscopy, ATP production and mitochondrial membrane potential measurements to assess mitochondrial abundance and mitochondrial activity of RA differentiated SH-SY5Y cells challenged with MPP(+). Our observations indicate that SH-SY5Y cells show a molecular phenotype profile that is characteristic for DAergic cells, and are an appropriate model to study the effects of PD-related mitochondrial inhibition on cell viability and function.

## Materials and Methods

### Cell Culture and MPP(+) Treatment

**Figure 1:**
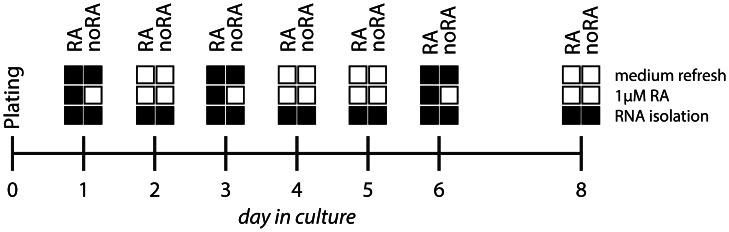
Culture setup of SH-SY5Y cells treated with or without RA. Cells were cultured for 8 days in medium only (the noRNA group) or in medium supplemented with 1 µM retinoic acid (the RA group). Cells were harvested for RNA isolation at the indicated time points.

Human SH-SY5Y neuroblastoma cells were obtained from The European Collection of Cell Culture (ECACC, 94030304, Sigma-Aldrich). Cells were cultured in Dulbecco’s Modified Eagle’s Medium (DMEM)/F-12 without L-glutamine (Invitrogen) supplemented with 0.5% fetal calf serum (FCS), 100 U/ml penicillin (Sigma) and 0.1 mg/ml streptomycin (Sigma) for 8 days at 37°C in 5% CO_2_. Cells were grown in 96-well plates coated with 0.1 mg/ml poly-L-lysine (PLL, Sigma) and 1 mg/ml growth factor reduced Matrigel Matrix without phenol red (BD Biosciences). 10,000 cells per well were plated. As we were interested in the effects of retinoic acid on the growth and differentiation of SH-SY5Y cells cultured cells were assigned to two treatment groups: culture in medium only (noRA) and culture with medium supplemented with 1 µM all trans-retinoic acid (RA, Sigma) (Figure 1).

### RNA Isolation and Quality Control

Cells were harvested for RNA isolation at the time points indicated in Figure 1. Cells were lysed by adding 50 µl of Trizol Reagent (Invitrogen) to each well, followed by incubation on ice for 10 min. Each time point/treatment condition consisted of 30 wells, divided into three replicates of 10 pooled wells. Phase separation was performed with chloroform and Phase Lock Gel (5 Prime). The final aqueous phase was diluted with an equal volume of 70% ethanol. RNA was isolated using RNeasy Micro columns (Qiagen) according to the manufacturer’s instructions. RNA quantity and purity were determined by NanoDrop ND-1000 spectrophotometer (Nanodrop Technologies) and RNA integrity was determined using the RNA Integrity Number (RIN) measured on an Agilent 2100 Bioanalyzer (Agilent Technologies).

### RNA Labeling and Microarray Hybridization

RNA samples were amplified and labeled with either Cy5-CTP or Cy3-CTP using the two-color Low Input Quick Amp Labeling Kit (Agilent Technologies) and purified with the RNeasy Micro kit according to the manufacturer’s protocol. Quality control was performed where cRNA quantity and dye integration were determined by NanoDrop measurement and cRNA fragment length was investigated with the Bioanalyzer.

Labeled cRNA was hybridized on Agilent 4×44K Whole Human Genome arrays (Agilent Technologies, Part Number G4112F) according to the manufacturer’s protocol. Briefly, 825 ng of Cy3-CTP or Cy5-CTP labeled cRNA was fragmented for 30 min at 60°C in 1×fragmentation buffer (Agilent Technologies) and loaded onto the array in 1×GEx Hybridization Buffer (Agilent Technologies). Arrays were incubated at 60°C in a rotating hybridization chamber for 17 h, after which they were washed in 6×SSPE 0.005% N- Lauroylsarcosine (Sigma-Aldrich) for 5 min and in 0.06×SSPE 0.005% N-Lauroylsarcosine for one minute. Finally, slides were washed in acetonitrile (Sigma-Aldrich) for 30 seconds and dried in a nitrogen flow. Microarrays were scanned in an Agilent DNA Microarray Scanner at 5 µm resolution at 10% and 100% PMT settings. Scan images were combined and quantified using Agilent Feature Extraction Software (version 9.5.1). The hybridization set up can be found in the Figure S1.

### Microarray Normalization and Single Gene Analysis

Raw expression data, generated by Feature Extraction software, was imported into the R statistical processing environment using the LIMMA package [42] in Bioconductor (http://www.bioconductor.org). All features for which one or more foreground measurements were flagged as a non-uniformity outlier or as a saturated outlier were excluded from further analysis. We previously demonstrated that the intensity-based analysis of complex ratio-based designs is more efficient and powerful than the standard ratio-based analysis [43]. We have therefore used an intensity-based analysis for this dataset. The individual signal intensities were extracted from the ratio measurements and 2log transformed intensity measurements were used for further analysis. Normalization between arrays was performed using the quantile algorithm in LIMMA. Due to the hybridization scheme, some samples were hybridized in duplicate - in this case the mean expression level of the duplicates was used.

To detect genes with differential expression between treatment conditions (RA versus noRA) and during the time of culture, a two-way analysis of variance (ANOVA) was performed on the 2log transformed intensity levels for each probe to detect features with a significant interaction between treatment and time in culture. All values were corrected for multiple testing using the Benjamini-Hochberg (BH) algorithm. All corrected p-values <0.05 were considered significant. The data discussed in this publication have been deposited in NCBI's Gene Expression Omnibus [44] and are accessible through GEO Series accession number GSE43368 (http://www.ncbi.nlm.nih.gov/geo/query/acc.cgi?acc=GSE43368).

### Cluster Analysis, Pathway Analysis and Transcription Factor Selection

To visualize the relationship between all treatment-time point combinations, unsupervised cluster analysis was performed on all genes with a significant change in expression (p<0.05) after BH correction using the heatmap function in R. Additionally, temporal profiles were created for the RA samples for each gene by: 1. normalizing expression values by subtracting the average expression of all measurements, and 2. averaging the expression for each treatment-time point condition. These temporal profiles were clustered into 20 clusters by a soft clustering approach based on fuzzy c-means using the M-Fuzz package (version 16.0) in Bioconductor to detect patterns of co-regulated genes over time for each treatment group. Clusters with up or down regulation of gene expression in time were chosen for further analysis using Ingenuity Pathways Analysis (IPA) and Gene Ontology GOstat software [45].

In order to further examine the RA differentiation process, all transcription factors (TFs) were selected from the significantly regulated gene list. TF genes were selected based on regular expression terms indicating a transcriptional function of the gene: "transcription, zinc.*f, EF.*hand, forkhead, homeo(.*domain|.*box), nucleic.*acid, nuclear(.*factor|.*receptor), DNA\Wbinding, Helicase, \Wets\W, Kruppel" [46]. All TF hits were further confirmed and their specific functional roles analyzed with the NCBI Entrez Gene database.

### Reverse Transcription and Quantitative PCR

180 ng of each RNA sample was reverse transcribed using the QuantiTect Reverse Transcription Kit (Qiagen). 1/250 or 1/300 of total cDNA was used for each qPCR reaction, depending on the expression level of each gene. In each reaction 3 pmol of forward and reverse primer (see Table S1 in Tables S1 for primer sequences) was used with 10 µl 2×SYBR green ready reaction mix (Applied Biosystems, Foster City, CA, USA), totaling a volume of 20 µl per reaction. Reactions were carried out on an ABI 7300 sequence system (Applied Biosystems). Each primer pair was checked for primer dimers by analyzing dissociation curves. As housekeeping genes we used the most stably expressed genes in the microarray dataset. Six genes were selected, based on their biological function, such as cytoskeleton or mitochondrial related, and tested with qPCR (SELK, PPP1R8, NDUFV2, NY-SAR-48, PRKCZ, UQCRFS1). With the use of GeNorm software [47] the final three most stable housekeeping genes were identified (NDUFV2, NY-SAR-48 and PRKCZ) and the normalization factor was calculated.

### Immunocytochemical Staining

Cells were cultured in 24-well plates on glass cover slips as described above and fixed with 4% paraformaldehyde (PFA) (Sigma) for 30 min followed by 30 min blocking at room temperature (RT) in blocking buffer (1×phosphate buffered saline (PBS), 0.5% Triton X-100 (Sigma), 0.25% gelatin (Merck) and 2% fetal calf serum (FCS). The following primary antibodies were applied in 1×PBS/2% FCS/0.25% gelatin/0.5% Triton X-100 (Sigma) and incubated at 4°C overnight: anti-tyrosine hydroxylase (TH) (rabbit, Institute Jacques Boy SA, Reims, France, 208020234) at 1∶200, anti-VMAT2 (rabbit, Millipore, AB1767) at 1∶200, and anti-dopamine at 1∶100 (rabbit [48]). Cells were washed and labeled with Alexa 488 anti- rabbit antibodies (Invitrogen, Carlsbad, CA, USA) at 1∶800 dilution for 2 h at RT in 1×PBS/2% FCS followed by 20 min nuclear staining with Hoechst 33258 (Molecular Probes; 10 mg/ml, diluted 1∶20,000 in H2O). Cells were mounted in Mowiol solution (0.1 M Tris pH 8.5, 25% glycerol, 10% w/v Mowiol 4–88 (Sigma)) and analyzed with the use of a confocal laser scanning microscope (Zeiss, Sliedrecht, The Netherlands).

### Protein Isolation and Western Blot Analysis

For Western blot analysis cells were cultured as described above in 24 well plates in two conditions: RA treated and noRA treated. On day 8, cells were incubated on ice for 10 min in 70 µl RIPA buffer (25 mM Tris-HCl pH 7.4 (Sigma), 150 mM NaCl (Sigma), 1% NP40 (AppliChemicals), 1% sodium deoxycholate (Sigma), 0.1% SDS and 1×Complete Protease Inhibitor (Roche)). The cell lysate was then collected, sonicated and the protein concentration was calculated with bicinchoninic acid protein assay kit (Pierce, Thermo Scientific). For Western blot analysis, each sample was heated in 5×loading buffer containing 10% sodium dodecyl sulphate (SDS, MP Biomedicals) and 5% ß-mercaptoethanol (Sigma) at 95°C for 5 min and loaded on 8% SDS gel. Tris-glycine sodium dodecyl sulphate polyacrylamide gel electrophoresis was performed using the BioRad Mini-PROTEAN 3 gel electrophoresis system (Bio-Rad Laboratories, Hercules, CA, USA) and proteins were semi-dry transferred to nitrocellulose membranes and blocked with 5% fat-free milk powder in 1×tris buffered saline (TBS)/0.05% Tween-20 (Sigma) for 1 h at room temperature. Blots were incubated with antibodies against DBH (sheep, Millipore, AB 1537) at 1∶200 and ß-actin (mouse, Sigma-Aldrich, A5316) at 1∶1000 at 4°C overnight in TBS/0/05% Tween-20. The primary antibodies were further detected, first with biotyn anti-sheep antibody (Vector laboratories) at 1∶400 dilution and then with SA-Cy5 (Jackson’s Lab) at 1∶800 for DBH detection, and anti-mouse IR-dye 800 at 1∶2000 for ß-actin detection. Blots were scanned and analyzed using the Odyssey Infrared Imager and Odyssey 2.1 scanning software (LI-COR biosciences). The ß-actin signal was used to normalize the final protein quantifications.

### Detection of Functional DAT and NAT

DAT and NAT activity in RA-differentiated SH-SY5Y cells were measured using different concentrations (0,003 µM–3.0 µM) of the selective DAT inhibitor 1-(2-[*bis*(4-fluorophenyl)methoxy] ethyl)-4-(3 phenylpropyl) piperazine (GBR 12909) or with different concentrations (0,0001 µM–0,1 µM) of the selective NAT inhibitor desipramine (DMI) in culture medium for 1 h as previously described [49]. In short, cultures were incubated for 20 min at 37°C with 1 mM [^3^H]DA in Krebs-Ringer buffer (16 mM sodium phosphate, 119 mM NaCl, 4.7 mM KCl, 1.8 mM CaCl2, 1.2 mM MgSO4, 1.3 mM EDTA, and 5.6 mM glucose; pH 7.4). Nonspecific uptake was measured in the presence of 10 µM mazindol. After washing three times with ice-cold Krebs-Ringer buffer, cells were lysed in 1 N NaOH and [^3^H] DA uptake was determined by liquid scintillation counting. Specific [^3^H] DA uptake was calculated by subtracting the amount of uptake measured in the presence of mazindol.

### Assessment of Mitochondrial Activity

#### High-content quantification of mitochondrial intensity

SH-SY5Y cells were cultured in the presence or absence of RA and treated with different concentrations of MPP(+). RA was added as indicated in Figure 1. MPP(+) was administered on day 3 and day 6 in culture. For mitochondrial abundance, cells were fixed after 8 days and incubated with 100 nM MitoTracker solution (Molecular Probes) diluted in 1×PBS for 20 min at RT. Cells were washed 2×5 min in 1×PBS, and nuclear staining was then performed by incubating cells in 100 µl Hoechst for 20 min at RT. Cells were washed 2×5 min in H_2_O and left in H_2_O for image collection. Image acquisition and analysis were performed using an ArrayScan VTI HCS Reader instrument (Thermo Scientific) and the Compartmental Analysis Bioapplication. Twenty images per well were collected, and mitochondrial activity was measured on a per cell basis quantifying both the number and the intensity of MitoTracker-positive spots in the cytoplasmic region of the cell. Readout from five wells was averaged and biological replicates were merged.

#### Mitochondrial membrane potential measurements

The mitochondrial membrane potential was measured in live cells using the Tetramethylrhodamine, Methyl Ester (TMRM) assay (Invitrogen, USA). On day 8 of culture, cells were incubated with TMRM at a final concentration of 450 nM for 20 min. After incubation, a final concentration of 1.4 g/L brilliant black (Sigma, Germany) was added and fluorescence was measured at ex 530/em 580 on a Varioskan Flash (Thermo Scientific). Twelve point well scans were performed and the average fluorescence intensity was calculated per well.

#### ATP production measurements

ATP production was measured on day 8 in culture using the Cellular ATP Kit HTS (BioThema, Handen, Sweden), according to the manufacturer’s recommendations. The luminescence levels were measured on a Varioskan Flash (Thermo Scientific). A 5 second integrated luminescent measurement was performed, and ATP concentrations were calculated after spiking and remeasurement of the cell lysates with a known concentration of ATP. Statistical analyses for all mitochondrial assays were performed using Student T-test. P values below 0.05 were considered significant.

## Results

### Retinoic Acid Induces Neuronal Differentiation of SH-SY5Y Cells

#### Gene expression changes induced by RA treatment

To study the effects of RA on the differentiation of SH-SY5Y cells we performed a microarray analysis of RA treated and untreated cells during an eight-day culture period. After hybridization, 2329 microarray probes (representing 2020 genes) showed differential gene expression in RA cells compared to no retinoic acid treated (noRA) cells (BH-corrected p<0.05, ANOVA-derived interaction between treatment and time in culture). The 50 most significantly regulated genes following RA treatment are listed in Table 1 (for all significantly regulated genes, see Table S2 in Tables S1). Real time quantitative PCR (qPCR) was used to validate the significant changes in expression of 10 selected genes as identified by microarray analysis (Table S1 in Tables S1, including sequences of used primers). Gene expression levels as measured by microarray and qPCR were highly correlated (see Table S1 in Tables S1 for correlation coefficient values).

**Table 1:** List of the 50 most significantly regulated genes in RA treated SH-SY5Y cells compared to noRA cells as identified by microarray analysis.

SystematicName	GeneName	Description	P.Value	Fold changes			Cluster
				RA D8/D1	noRA D8/D1	D8 RA/noRA	
NM_002923	RGS2	Homo sapiens regulator of G-protein signalling 2, 24 kDa	1.10E-09	12.43	0.98	13.90	4
NM_017414	USP18	Homo sapiens ubiquitin specific peptidase 18	1.10E-09	0.42	4.00	0.13	13
NM_152654	DAND5	Homo sapiens DAN domain family, member 5	1.10E-09	0.14	2.67	0.04	13
NM_001005339	RGS10	Homo sapiens regulator of G-protein signalling 10, transcript variant 1	3.73E-09	0.19	0.89	0.23	13
NM_001496	GFRA3	Homo sapiens GDNF family receptor alpha 3	3.73E-09	0.26	3.89	0.05	13
NM_001008540	CXCR4	Homo sapiens chemokine (C-X-C motif) receptor 4, transcript variant 1	6.13E-08	0.19	0.74	0.30	13
NM_139314	ANGPTL4	Homo sapiens angiopoietin-like 4, transcript variant 1	6.70E-09	4.57	0.18	24.65	16
NM_018712	ELMOD1	Homo sapiens ELMO/CED-12 domain containing 1	6.70E-09	22.20	0.99	19.34	3
NM_000599	IGFBP5	Homo sapiens insulin-like growth factor binding protein 5	9.92E-09	0.10	0.22	0.44	13
NM_033066	MPP4	Homo sapiens membrane protein, palmitoylated 4 (MAGUK p55 subfamily member 4)	1.24E-08	7.11	0.80	8.92	3
NM_002247	KCNMA1	Homo sapiens potassium large conductance calcium-activated channel, subfamily M, alpha member 1, transcript variant 2	1.24E-08	0.13	3.02	0.05	13
NM_005461	MAFB	Homo sapiens v-maf musculoaponeurotic fibrosarcoma oncogene homolog B (avian)	1.24E-08	2.41	0.39	5.89	16
NM_002166	ID2	Homo sapiens inhibitor of DNA binding 2, dominant negative helix-loop-helix protein	2.24E-08	0.77	0.30	3.00	17
AF131762	AF131762	Homo sapiens clone 25218 mRNA sequence	2.33E-08	5.39	0.75	6.12	3
AK091547	LOC730125	Homo sapiens cDNA FLJ34228 fis, clone FCBBF3025417	2.33E-08	0.08	0.76	0.13	13
NM_152703	SAMD9L	Homo sapiens sterile alpha motif domain containing 9-like	2.49E-08	5.26	0.55	7.06	4
AK125162	AK125162	Homo sapiens cDNA FLJ43172 fis, clone FCBBF3007242	2.60E-08	0.08	0.52	0.18	5
NM_001419	ELAVL1	Homo sapiens ELAV (embryonic lethal, abnormal vision, Drosophila)-like 1 (Hu antigen R)	3.21E-08	0.31	1.23	0.33	5
A_23_P170719	A_23_P170719	Unknown	3.21E-08	4.25	0.31	13.74	16
NM_000905	NPY	Homo sapiens neuropeptide Y (NPY)	3.21E-08	0.03	0.69	0.05	5
NM_002849	PTPRR	Homo sapiens protein tyrosine phosphatase, receptor type, R, transcript variant 1	3.66E-08	2.12	0.85	2.93	14
THC2656519	THC2656519	Unknown	3.66E-08	0.20	0.94	0.27	13
CR618615	CR618615	full-length cDNA clone CL0BB018ZH05 of Neuroblastoma of Homo sapiens (human)	4.16E-08	0.12	0.82	0.15	5
NM_197966	BID	Homo sapiens BH3 interacting domain death agonist, transcript variant 1	4.49E-08	0.28	1.04	0.29	13
NM_007124	UTRN	Homo sapiens utrophin	4.92E-08	0.36	2.87	0.18	13
NM_016354	SLCO4A1	Homo sapiens solute carrier organic anion transporter family, member 4A1	4.99E-08	0.10	1.24	0.07	13
NM_006157	NELL1	Homo sapiens NEL-like 1 (chicken)	4.99E-08	0.22	5.58	0.05	13
NM_024817	THSD4	Homo sapiens thrombospondin, type I, domain containing 4	5.72E-08	15.75	0.80	19.77	3
NM_003713	PPAP2B	Homo sapiens phosphatidic acid phosphatase type 2B, transcript variant 1	5.72E-08	0.46	7.42	0.08	2
NM_003385	VSNL1	Homo sapiens visinin-like 1	6.08E-08	0.26	4.10	0.09	13
NM_018286	TMEM100	Homo sapiens transmembrane protein 100	6.85E-08	0.25	1.62	0.20	13
NM_004598	SPOCK1	Homo sapiens sparc/osteonectin, cwcv and kazal-like domains proteoglycan (testican) 1	7.77E-08	0.53	8.70	0.07	2
BX648591	BX648591	Homo sapiens mRNA; cDNA DKFZp686G14198 (from clone DKFZp686G14198)	7.90E-08	0.29	1.32	0.25	5
NM_000507	FBP1	Homo sapiens fructose-1,6-bisphosphatase 1	9.46E-08	0.18	1.17	0.18	13
NM_001034852	SMOC1	Homo sapiens SPARC related modular calcium binding 1, transcript variant 1	1.00E-07	3.71	1.31	4.74	4
NM_004982	KCNJ8	Homo sapiens potassium inwardly-rectifying channel, subfamily J, member 8	1.08E-07	0.25	1.33	0.19	5
NM_015150	RFTN1	Homo sapiens raftlin, lipid raft linker 1	1.08E-07	0.46	3.74	0.13	11
NM_003068	SNAI2	Homo sapiens snail homolog 2 (Drosophila)	1.22E-07	2.20	0.32	7.42	16
NM_024645	ZMAT4	Homo sapiens zinc finger, matrin type 4	2.37E-07	0.66	0.67	1.28	11
NM_003836	DLK1	Homo sapiens delta-like 1 homolog (Drosophila), transcript variant 1	2.37E-07	0.07	1.43	0.05	13
NM_000087	CNGA1	Homo sapiens cyclic nucleotide gated channel alpha 1	2.37E-07	4.60	0.70	5.18	4
NM_001797	CDH11	Homo sapiens cadherin 11, type 2, OB-cadherin (osteoblast)	2.37E-07	1.48	0.86	2.01	18
ENST00000284894	NCAM2	Neural cell adhesion molecule 2 precursor (N-CAM 2)	2.37E-07	24.28	1.78	12.63	3
NM_000693	ALDH1A3	Homo sapiens aldehyde dehydrogenase 1 family, member A3	2.37E-07	0.14	1.23	0.14	13
THC2637644	THC2637644	Unknown	2.37E-07	0.16	0.55	0.31	13
NM_002615	SERPINF1	Homo sapiens serpin peptidase inhibitor, clade F (alpha-2 antiplasmin, pigment epithelium derived factor), member 1	2.37E-07	0.27	0.94	0.26	13
NM_000845	GRM8	Homo sapiens glutamate receptor, metabotropic 8	2.37E-07	0.46	2.60	0.20	11
NM_145008	YPEL4	Homo sapiens yippee-like 4 (Drosophila)	2.37E-07	4.33	1.21	3.45	4
NM_078481	CD97	Homo sapiens CD97 molecule, transcript variant 1	2.37E-07	0.20	1.07	0.23	13
BC045679	LOC285878	Homo sapiens hypothetical protein LOC285878, mRNA (cDNA clone IMAGE:5299807)	2.37E-07	6.94	0.88	7.55	3

Gene expression fold changes are given for the comparison of RA treated cells on day 8 (D8) versus RA day 1 (D1), noRA D8 versus noRA D1 and RA D8 versus noRA D8. The last column indicates the cluster number to which that gene is assigned based on its expression profile (see Figure S2). CXCR4 and CR618615 were represented by more than one probe in the list and the corresponding p-values and fold changes were averaged. P-values are Benjamini-Hochberg corrected. The complete list of all significantly regulated genes can be found in Table S2 in Tables S1.

IPA confirmed that RA induces the retinoic acid receptor (RAR) signaling pathway, as the significantly regulated genes in RA cells are highly enriched for members of this pathway (p = 0.013) (Figure S2). For example, RAR-A and -B, which control all RA induced signaling, are highly upregulated following RA treatment (fold change D8 RA/noRA 2.79 and 23.45 respectively).

#### RA regulates cellular differentiation and proliferation

**Figure 2:**
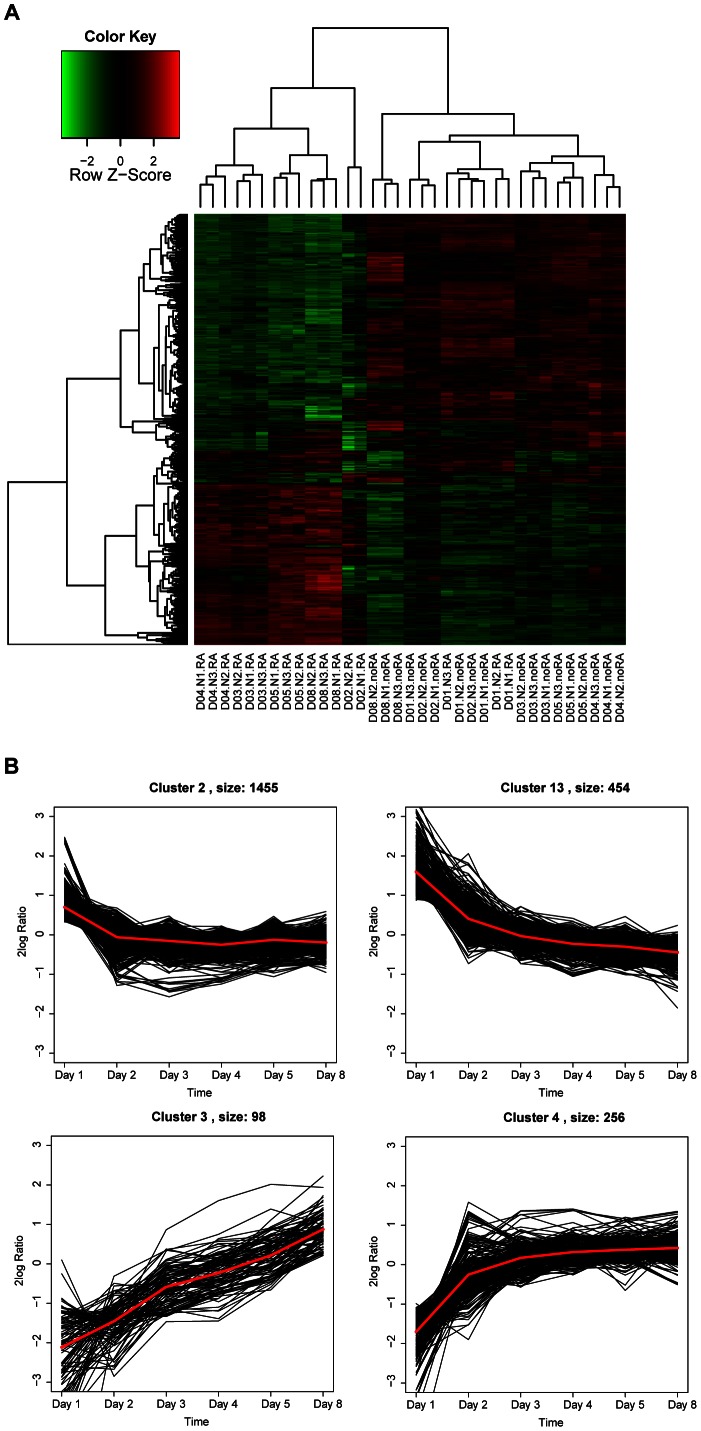
Regulation of gene expression in RA treated cells. **A.** Heat-map of all significantly regulated genes indicates that differences in gene expression can be used to separate both treatment condition and time in culture. Day of culture, the biological replicate, and the culture conditions are indicated on the X-axis. On the Y-axis each row represents one gene. Green indicates downregulation, and red indicates. **B.** The 4 clusters with the most distinct pattern of gene regulation in time in RA treated cells (for all clusters, see Figure S3). Each line represents one gene. The red line represents the average pattern of expression of all genes in the cluster. Clusters sizes are indicated next to the cluster numbers.

Unsupervised hierarchical cluster analysis of the expression levels of all significant genes showed a clear distinction based on treatment condition and time in culture (Figure 2A). Except for Day 1, the RA treatment induced a highly distinct gene expression program on which treatments can be separated. Additionally, in RA treated cells, we observed distinct expression differences between the early time points (days 3 and 4) and later time points (days 5 and 8), indicating that the time of culture played a role in RA induced expression changes. To a lesser extent, time of culture also determined the gene expression profile of cells not treated with RA, with days 1 and 2 clustering together and days 3, 4 and 5 forming a distinct cluster. Day 8 stands separate. In general, gene expression changes are less pronounced during time in culture in noRA cells as compared to RA treated cells.

We also performed a gene expression cluster analysis of the temporal patterns that emerged over time of culturing for all detectable genes in RA treated cells. Four clusters (Figure 2B; clusters 2, 13, 3 and 4) out of a total of 20 clusters (Figure S3) showed a clear directional pattern of expression, with genes in clusters 2 and 13 downregulated over time and genes in clusters 3 and 4 upregulated over time. Gene Ontology analysis (GOstat) and IPA were performed to determine which biological processes are overrepresented in these temporal clusters (Table S3 in Tables S1). These biological processes can be grouped into five functional categories: development, cellular development, neuronal function, proliferation and cell death.

#### RA induces the expression of transcription factors that regulate neuronal differentiation

To investigate the molecular mechanisms by which RA alters cellular development, we examined the effects of RA on the expression of transcription factors (TFs). We identified 103 significantly regulated TFs in RA-treated cells. Using gene function references derived from the NCBI Gene and IPA databases, we aimed at uncovering the biological function of each TF (Table S4 in Tables S1). We found 74 TFs with known functions. Out of these 74 TFs, 20 regulate cellular development and differentiation. Most of these were positive regulators of differentiation, and three are both positive and negative regulators of cellular differentiation i.e. ID1 (RA/noRA 2.79), SP1 (RA/noRA 1.45) and ZNF521 (RA/noRA 3.39). At day 8, 13 pro-differentiation TFs were upregulated in RA treated cells as compared to untreated cells. Examples of upregulated pro-differentiation TFs include ALX3 (fold change RA/noRA 1.42), KLF13 (RA/noRA 2.43) and NR4A3 (RA/noRA1.6). In contrast, only 4 pro-differentiation TFs are downregulated in RA treated cells, i.e. ELF4 (RA/noRA 0.25), SIX6 (RA/noRA 0.41), E2F5 (RA/noRA 0.42) and RUNX1 (RA/noRA 0.45).

In addition to the predominant upregulation of TFs that promote general cellular differentiation, RA alters the expression of 13 TFs that are known to specifically stimulate neuronal development/differentiation and function. RA treatment increases the expression of 6 positive regulators of neuronal development and differentiation i.e. NCOA7 (RA/noRA 3.97), TLX2 (RA/noRA 2.79), ID3 (fold change RA/noRA 2.11), NFE2L2 (RA/noRA 1.68), ZNRF1 (RA/noRA 1.27) and HOXD10 (RA/noRA 3.30). Moreover, 4 negative regulators of neuronal differentiation and function are downregulated after RA treatment i.e. TFAP2B (RA/noRA 0.37), ISL1 (RA/noRA 0.56), SIX3 (RA/noRA 0.41) and ATF5 (RA/noRA 0.55). Only one neuronal differentiation and neuronal survival promoting TF, MEF2C, is downregulated after RA treatment (RA/noRA 0.63).Two TFs that are responsible for neuronal differentiation are downregulated during the time of culture independently of RA treatment i.e. MSX2 (RA/noRA 0.92) and PHOX2B (RA/noRA 0.92).

We also observed RA-induced changes in the expression of TFs that are involved in regulating apoptosis and cell death. 4 pro-apoptotic TFs were upregulated and 3 were downregulated after RA treatment. Finally, 14 TFs involved in cellular proliferation were also changed after RA treatment. 6 pro-proliferation TFs were upregulated and 7 were downregulated. CGREF1, a negative regulator of proliferation, was also downregulated (RA/noRA 0.49). The functional implications of these expression changes of TFs involved in apoptosis and proliferation are not straightforward. The specific functions and differential expression patterns of all TFs mentioned above can be found in Table S4 in Tables S1.

Taken together, the TF expression profiles indicate that RA treated SH-SY5Y cells are in a pro-differentiation transcriptional state and, more specifically, a differentiation state towards a neuronal phenotype. Additionally, during differentiation specific changes in the expression of apoptotic and proliferative TFs are observed.

### Retinoic Acid Treated SH-SY5Y Cells Show a DAergic-like Neurotransmitter Phenotype

#### Expression of early DAergic markers in SH-SY5Y cells

**Figure 3:**
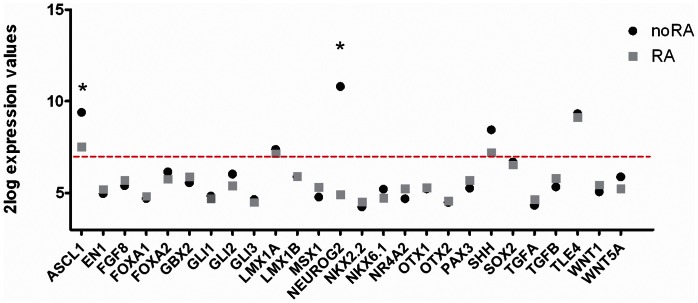
Expression of early stage dopaminergic markers. Based on Smidt and Burbach [50], expression of early stage DAergic markers in noRA (black circle) and RA (gray square) differentiated SH-SY5Y cells on culture day 8 as measured by microarray analysis. The red line represents the cutoff between detected gene expression (2log expression >7) and undetected gene expression (2log expression ≤7). Significantly regulated genes between the two conditions are marked with an ‘*’.

We were particularly interested in characterizing the putative DAergic cell phenotype of RA treated SH-SY5Y cells. To address this, we investigated the expression profiles of genes typically required for DAergic neuronal development *in vivo*. We discriminated between markers that are expressed either during early or late stages of DA cell development based on a study by Smidt and Burbach [50]. These authors discuss the specific cascades of molecular codes that regulate the generation of mesodiencephalic DAergic neurons in the developing mouse brain. During the early stage of DAergic neuron development two types of signals are required, i.e. external inductive signals such as growth factors present in the environment (such as FGF, TGF and WNT), and intrinsic TFs expressed by the cells themselves. At 8 days in culture, the microarray analysis established that SH-SY5Y cells express 5 out of the 26 markers that have a role in the early stage of DAergic neuronal development (Figure 3). Two of these early stage markers were significantly downregulated after RA treatment (fold change on day 8 between RA and noRA: ASCL1 0.27 and NEUROG2 0.02), but overall RA treatment did not significantly affect the expression of these markers. In addition to the microarray data, we have confirmed the expression of these markers with qPCR (Table S1 in Tables S1). We conclude that SH-SY5Y cells do not express early stage DAergic markers, irrespective of culture conditions.

#### SH-SY5Y cells synthesize DA

**Figure 4:**
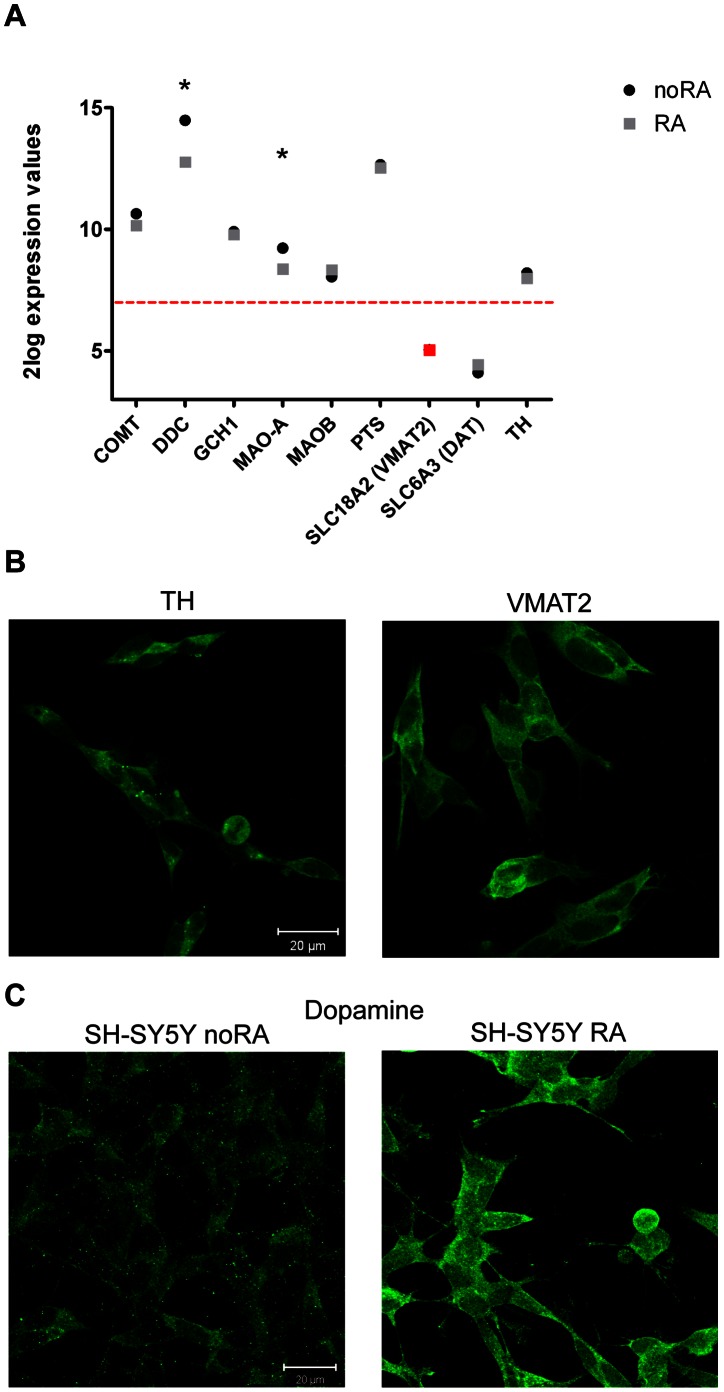
Expression of dopamine synthesis and turnover markers in noRA and RA differentiated SH-SY5Y cells at culture day 8. Selection of genes is based on NCBI Gene data base and IPA. **A.** Microarray expression values of these molecular markers are presented for the noRA (black circle) and RA (gray square) treated cells at D8. Red line characterizes the cut-off at 2log intensity of 7 demarcating expression versus no expression of the mRNA in these cells (equivalent to 2×background levels). SLC18A2 (VMAT2) was not detected on the microarray, but confirmed to be expressed by qPCR analysis (red marking). Significantly regulated genes between the two conditions are marked with an ‘*’. **B.** Immunocytochemistry of two dopamine synthesis and transport proteins TH and VMAT2 expressed by the RA treated cells at D8. **C.** Immunocytochemical detection of DA in noRA and RA SH-SY5Y cells. Images, taken with the same laser intensity, consist of a z-stack projection of the cell layer.

Mature DAergic cells should produce DA and express molecules that are important for DA synthesis, transport and turnover. SH-SY5Y cells express all of the DA synthesizing enzymes and DA degrading enzymes as detected by micorarray analysis (Figure 4A) and by qPCR (Table S1 in Tables S1). Although the vesicular monoamine transporter (VMAT2) was initially not detected by microarray analysis, qPCR confirmed its presence in RA-treated cells. However DAT is not detected in these cells, also not with qPCR. Interestingly, the DA, serotonin and tryptamine synthesizing enzyme dopa decarboxylase (DDC) and dopamine breakdown enzyme MAO-A were both significantly downregulated by RA (RA/noRA fold change 0.30 and 0.55 respectively) (Figure 4A), but still highly expressed by these cells. We did not observe significant changes between RA and noRA cells in the expression levels of other DA synthesis and turnover markers such as COMT, GCH1, MAO-B, PTS, TH and SLC18A2 (VMAT2). In RA differentiated SH-SY5Y cells the expression of VMAT2 and TH, two genes with a central role in DA synthesis and transport, was confirmed at the protein level (Figure 4B). Thus, SH-SY5Y cells express all necessary molecules to produce DA, mostly independent of RA treatment.

We then asked whether SH-SY5Y cells produce DA and whether DA levels are altered in RA-treated SH-SY5Y cells. Immunohistochemical analysis of DA content showed that both noRA and RA treated cells produce DA and that DA immunoreactivity was increased following RA treatment (Figure 4C). As shown before, both noRA and RA treated cells show similar expression levels of DA synthesizing enzymes, with the exception of DDC and MAO-A. The decrease in MAO-A levels may explain the higher DA levels in RA treated cells. Our data clearly show that RA differentiated SH-SY5Y cell synthesize and store DA, and thus have a DAergic-like phenotype.

#### Expression of mature DAergic markers and neurotransmitter receptors in SH-SY5Y cells

**Figure 5:**
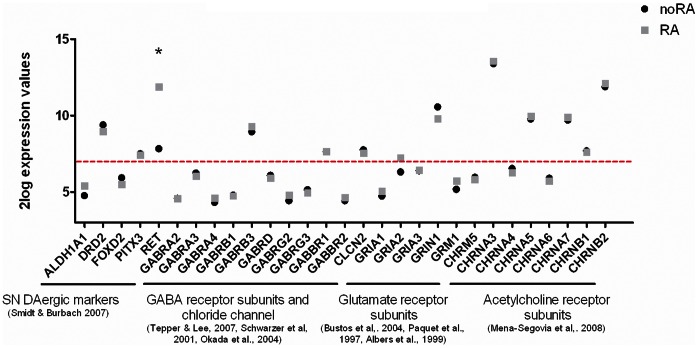
Expression of mature SN pars compacta DAergic neuron markers and neurotransmitter receptors. Gene levels are indicated in noRA (black circle) and RA (gray square) differentiated SH-SY5Y cells on culture day 8 as measured by microarray analysis. Graph shows expression of DAergic markers known to be expressed by mature SN DAergic neurons [50], and neurotransmitter receptors known to be expressed by SN DAergic neurons such as GABA receptor subunits and chloride chanels [51]–[53], glutamate receptor subunits [54]–[56] and nicotinic and muscaric acetylcholine receptor subunits [57]. Red line characterizes the cut-off at 2log intensity of 7 determining the expression and no expression of the mRNA in these cells (equivalent to 2×background levels). Significantly regulated genes between the two conditions are marked with an ‘*’.

Mature DAergic neurons in the brain express specific mature DAergic markers as well as specific neurotransmitter receptors. We compiled a list of these mature DAergic markers and identified the specific neurotransmitter receptor subunits based on literature (Figure 5) [50]–[56], [57]. In general, there is no difference in expression of the selected markers and receptors between noRA and RA differentiated SH-SY5Y cells, with one expection. Receptor tyrosine kinase (RET), expressed in mature DAergic neurons *in vivo*, was highly upregulated after RA treatment (RA/noRA fold change 16.13). SH-SY5Y cells further express 3 out of 5 mature DAergic markers, including DA receptor 2 (DRD2), irrespective of the culture conditions.

With respect to other neurotransmitter receptors, both noRA and RA SH-SY5Y cells express 2 out of 10 GABA receptor subunits known to be expressed by SN DAergic neurons, i.e. GABA _A_ β-3 subunit (GABRB3) and GABA _B_ 1 subunit (GABBR1). Interestingly, GABA _B_ receptors are expressed by DAergic neurons *in vitro*, whereas their expression *in vivo* is controversial [51]–[53]. Additionally, SH-SY5Y cells express a SN pars compacta specific chloride channel, CLCN2, which is necessary for GABA neurotransmission, but not the SN pars reticulata potassium-chloride co-transporter KCC2 (SLC12A5, data not shown, [51]).

SH-SY5Y cells further express 2 out of the 5 glutamate receptor subunits known to be expressed by SN DAergic neurons i.e. AMPA selective glutamate receptor 2 subunit (GRIA2), and NMDA selective glutamate receptor 1 subunit (GRIN1) [54]–[56].

Finally, both noRA and RA differentiated SH-SY5Y cells express 5 out of the 8 acetylcholine (ACh) receptor subunits known to be present in SN DAergic neurons. These include 3 out of the 5 subunits of nicotinic AChRα and 2 subunits of nicotinic AChRβ [57]. Muscarinic AChR5 showed either very low or no expression in SH-SY5Y cells.

In conclusion, SH-SY5Y cells express 45% of the known mature DAergic markers and neurotransmitter receptors characteristic for SN DAergic neurons. The expression pattern of these genes is independent of RA treatment.

#### RA alters the expression of DA receptor signaling components

Complementary to the analysis of classical DAergic markers, IPA revealed that RA treatment had a significant effect on genes playing a role in the DA receptor signaling pathway (p = 2.11E-02). The dopamine receptor inhibitor and desensitization protein NCS1 (also known as FREQ, fold change RA/noRA 0.62) was downregulated after RA treatment, providing a potential mechanism for enhanced DA signaling via the DRD2 receptor, which is expressed by SH-SY5Y cells (Figure 5). Indeed, RA decreases the expression levels of two adenylate cyclases that normally convert ATP to cAMP and are inhibited by DRD2 receptor activation (ADCY1 RA/noRA 0.61, ADCY7 RA/noRA 0.68). The cAMP dependent protein kinase regulatory unit 1 is also downregulated in the RA treated cells (PRKAR1B RA/noRA0.69).

In contrast, several protein phosphatase 2 subunits (a downstream target of DRD/AC/cAMP/PKA signaling pathway), were upregulated in RA treated cells: PPP2R5A (RA/noRA 1.59), PPP2R2B (RA/noRA 2.0) and PPP2R5B (RA/noRA 1.92, Table S2 in Tables S1). Interestingly, the regulatory B’beta subunits (PPP2R2B and PPP2R5B) are known to be present in the DAergic neurons and dephosphorylate TH [58]. The expression of several protein phosphatase 1 subunits, also downstream targets of the DRD/AC/cAMP/PKA signaling pathway, was also altered after RA treatment (PPP1R3C RA/noRA 2.53 and PPP1R14A RA/noRA 0.35).

### Effects of RA Treatment on Neurotransmitter Phenotypes Other than DA in SH-SY5Y Cells

**Figure 6:**
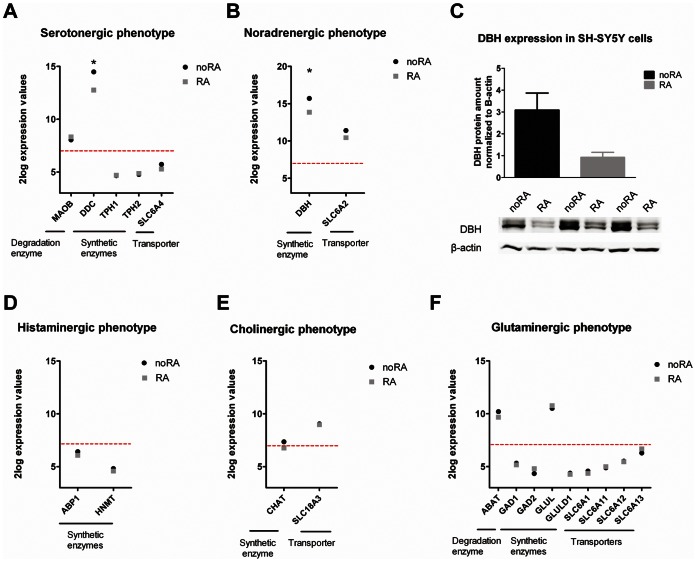
Expression of genes involved in five different neurotransmitter phenotypes. Genes involved in neurotransmitter phenotypes are indicated in undifferentiated (noRA) and RA differentiated (RA) SH-SY5Y cells for: serotonin (**A**), noradreneline (**B**), histamine (**D**), acetylcholine (**E**) and glutamate (**F**) phenotype. Genes below the red line are not expressed (microarray expression levels below 2×background). Significantly regulated genes between the two culture conditions are marked with an ‘*’. **C**. DBH protein expression was detected in three independent undifferentiated (noRA) and differentiated (RA) SH-SY5Y cell cultures by immunoblotting. Paired Student T-test showed a trend of decrease of DBH protein when normalized to the β-actin protein levels (p = 0.07).

We then investigated the expression of non-DA neurotransmitter markers in RA differentiated SH-SY5Y cells. Based on reviews by Verney [59] and Ernsberger [60], combined with NCBI Gene database annotations, we compiled a list of key non-DA neurotransmitter markers that are required for successful neurotransmitter production and transport, including synthesizing enzymes, degrading enzymes and neurotransmitter transporters (Figure 6). RA treatment results in downregulation of DDC and DBH (70% and 72% reduction compared to noRA cells), involved in serotonin and noradrenaline synthesis respectively. Other markers for histaminergic, cholinergic and glutaminergic neurotransmitter phenotypes are not altered in their expression after RA treatment. Serotonin production is probably inhibited by decreased expression of DDC, which, apart from DA production, is also involved in serotonin biosynthesis. Noradrenaline production may also be suppressed by decreased expression of DBH synthesizing enzyme, which we also validated on the protein level, where RA treated cells show a trend toward a significant 70% reduction of DBH protein levels (Figure 6C, p = 0.07). Most of the histamine, acetylcholine, and glutamate synthesis genes were undetectable in SH-SY5Y cells as measured by microarray, suggesting that these neurotransmitter phenotypes are not prominent in SH-SY5Y cells.

### The Effects of MPP(+) Toxicity on SH-SY5Y Cells

**Figure 7:**
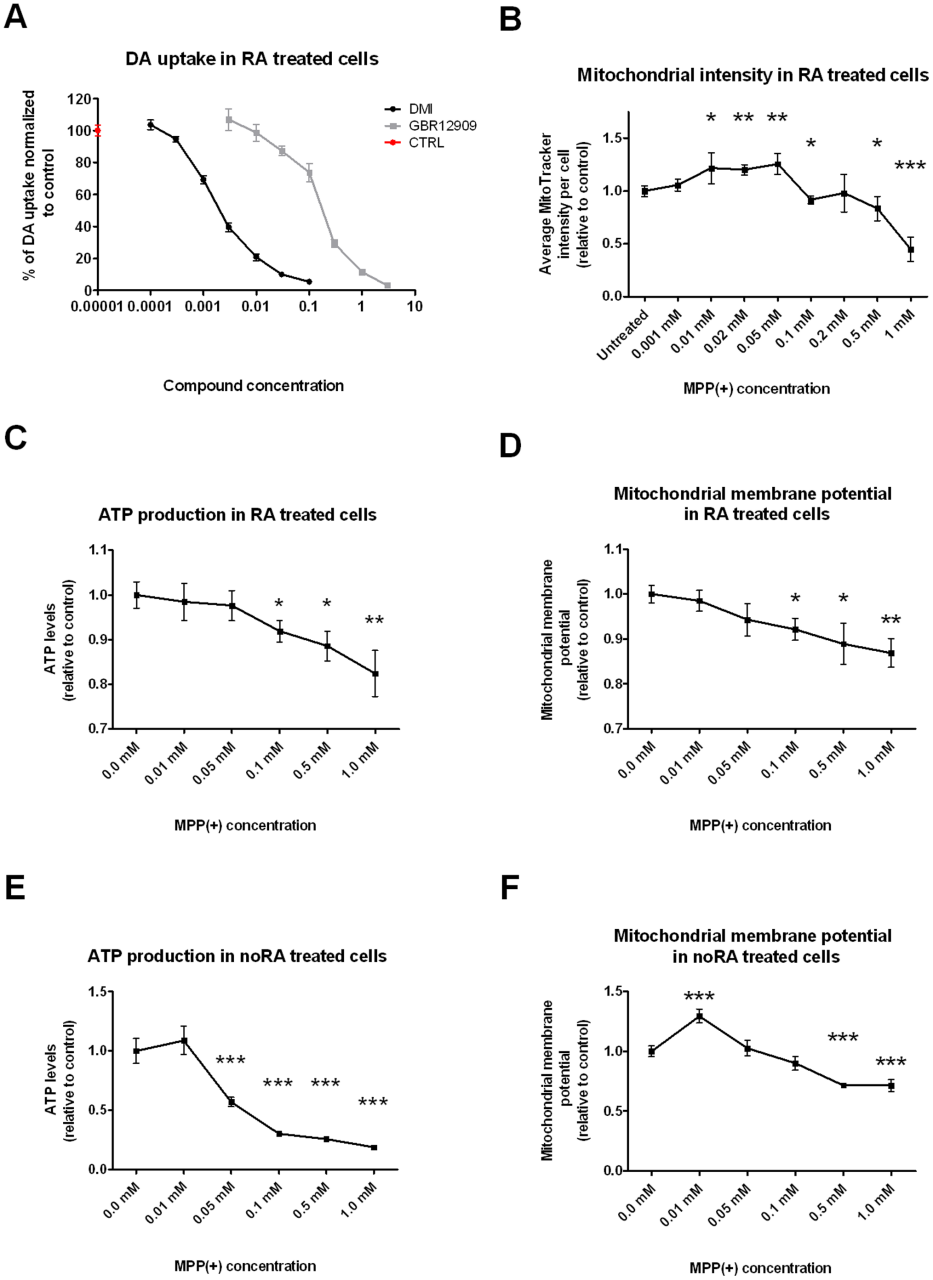
DAT and NAT activity and the effects of MPP(+) on mitochondrial activity in SH-SY5Y cells. **A.** Transport of radioactive DA in the presence of GBR12909 (selective DAT inhibitor) or DMI (selective NAT inhibitor) suggests that both of these transporter proteins are expressed and active in RA treated SH-SY5Y cells. **B.** Dose-response curve of MitoTracker intensity in RA differentiated SH-SY5Y cells treated with MPP(+). MitoTracker intensity was calculated by averaging the normalized mean cytoplasmic total intensity, mean cytoplasmic average intensity and mean cytoplasmic spot total intensity. Mitochondrial intensity is significantly increased in RA treated SH-SY5Y cells treated with 0.01 mM MPP(+) up to 0.05 mM, and significantly decreased in cells treated with 0.1 mM, 0.05 mM and 1 mM MPP(+). ATP levels were measured using Cellular ATP Kit HTS assay in RA (**C**) and noRA (**E**) treated SH-SY5Y cells treated with increasing MPP(+) concentrations. RA cells showed a significant decrease of ATP production starting from 0.1 mM MPP(+) treatment, whereas noRA cells showed a significant decrease in ATP production already at 0.05 mM MPP(+). Mitochondrial membrane potential was measured with TMRM assay in RA (**D**) and noRA (**E**) treated cells under different MPP(+) concentrations. RA cells showed a decrease in membrane potential at 0.1 mM up to 1 mM MPP(+), whereas noRA cells first showed an increase in membrane potential at 0.01 mM MPP(+) followed by a decrease at 0.5 and 1 mM MPP(+). All statistical analysis was performed using Student T-test (*p value <0.05, **p value <0.01, ***p value <0.001).

MPP(+) uptake by neurons requires expression of either the DAT or the NAT [36]. We therefore measured DAT and NAT activity in SH-SY5Y cells by selectively blocking these transporters using vanoxerine (BBR-12909, a DAT inhibitor) or desipramine (DMI, a NAT inhibitor), and measuring DA uptake. Both compounds decreased DA uptake in a dose-dependent manner (Figure 7A), indicating that both transporters are expressed and could therefore mediate MPP(+) uptake. NAT activity was significantly higher than DAT activity, which is in line with the observed mRNA levels (see Figure 6B for NAT expression (SLC6A2) and Figure 4A for DAT expression (SLC6A3)).

We then investigated whether MPP(+) could induce mitochondrial stress in RA differentiated SH-SY5Y cells by determining mitochondrial abundance (using MitoTracker intensity levels) and mitochondrial activity (by measuring ATP production levels and mitochondrial membrane potential) (Figure 7B, C and D). MitoTracker intensities were increased in MPP(+) doses from 0.01 mM to 0.05 mM in RA treated SH-SY5Y cells, whereas doses of 0.1 mM and above (except for 0.2 mM) decreased mitochondrial labeling in a dose-dependent manner when compared to MPP(+) untreated cells (Figure 7B). ATP production and mitochondrial membrane potential were also decreased in the RA treated SH-SY5Y cells at 0.1 mM MPP(+) (Figure 7C and D). Increased concentration of MPP(+) further decreased ATP production and mitochondrial membrane potential in a dose-dependent manner.

Finally, noRA cells were also sensitive to MPP(+) induced mitochondrial stress. They showed a much larger decrease in ATP production compared to RA cells with effects already seen at 0.05 mM of MPP(+). This ATP production was further impaired with increased MPP(+) concentrations (Figure 7E). Mitochondrial membrane potential on the other hand was only significantly decreased at 0.5 mM and 1 mM MPP(+) (Figure 7F).This indicates that mitochondria in noRA cells are also sensitive to the effects of MPP(+).

## Discussion

By combining genome-wide transcriptional profiling, gene ontology and IPA analysis of transcription factor expression and the expression of key markers associated with neurotransmitter phenotypes, we show that RA treatment of SH-SY5Y cells induces a differentiation program that promotes a general neuron-like state and predominant DAergic characteristics. MPP(+) treatment of RA differentiated SH-SY5Y cells decreases mitochondrial activity in a dose-dependent manner. This study thus extensively describes the molecular changes induced by RA treatment in SH-SY5Y cells and demonstrates that MPP(+) can be used to model PD-associated mitochondrial dysfunction *in vitro*.

### RA Treatment of SH-SY5Y Cells Initiates Transcriptional Changes that Promote a General Neuron-like Phenotype

RA is a key signaling molecule that has been shown to promote neuronal differentiation and to maintain a neuron phenotype through activation of retinoic acid receptors and their downstream targets [61]. Here, we provide an extensive characterization of the transcriptional events downstream of the RA signaling pathway in SH-SY5Y cells. GO analysis revealed that GO classes related to neuronal function were both enriched in the up- and downregulated gene clusters. However, a closer inspection of these data shows that the number of genes annotated with a neuronal function in the upregulated clusters (n = 115, or 35% of all upregulated genes) is much larger than the amount of neuronal genes in the downregulated clusters (71 genes, or 4% of all downregulated genes) (see Table S3 in Tables S1). Examples of upregulated genes important for neuronal differentiation include NCAM2 (12-fold upregulated on day 8), a cell adhesion molecule involved in neuronal compartmentalization [62], the BDNF receptor NTRK2 (27-fold higher on day 8), and NTNG2 (10-fold upregulated at day 8), a regulator of lamina-specific subdendritic compartmentalization [63]. Furthermore, several downregulated genes involved in neuronal function actually inhibit neuronal differentiation. Notable examples include MYCN, a positive regulator of S-phase reentry of neurons [64] and BMP7, a secreted signaling molecule that decreases neurogenesis of olfactory receptor neurons [65]. We also observed a downregulation of ASCL1, a gene that directly controls successive steps of neurogenesis and promotes proliferation of neuronal progenitors [66], and has been reported to be downregulated in SH-SY5Y cells after RA treatment [31].

The shift towards neuronal differentiation, as indicated by GO analysis, is further supported by our TF analysis, which shows that out of the 13 regulated TFs with a function in neuronal differentiation, 6 positive regulators of neuronal differentiation are upregulated, whereas 4 out of 5 negative regulators of neuronal differentiation are downregulated. Thus, RA alters the expression of 10 out of 13 neuronal differentiation TFs in such a way that neuronal differentiation is promoted.

Finally, our gene expression data corroborate the RA-induced upregulation of multiple genes already known to play a crucial role in neuronal differentiation of SH-SY5Y cells. These include NRF2, a transcription factor regulating the endogenous antioxidant response and neuronal differentiation [67], integrin α1 and ß1, cell membrane receptors involved in cell adhesion and recognition [28], and the Rho GTPase RAC1, a neurite outgrowth initiator [29]. Taken together, these data provide strong evidence for a pro-neuronal differentiation process in SH-SY5Y cells after treatment with RA.

### RA Differentiated SH-SY5Y Cells Express Multiple Markers Characteristic for SN DAergic Neurons in vivo, and Produce High Levels of DA

The precise neuron-like phenotype of RA differentiated SH-SY5Y cells has been controversial. Although some studies claim that RA increases the expression of TH [33], we and others report that TH is already present in non-differentiated cells and that RA-induced differentiation does not induce changes in TH protein levels [21]. To gain more insight into the exact DAergic characteristics of RA-differentiated SH-SY5Y cells, we investigated the expression of other markers that are associated with early development of DA neurons, DA synthesis and mature SN DAergic neurons. This analysis revealed that only 19% of the early stage DAergic markers are expressed by RA treated SH-SY5Y cells. On the other hand, all genes required for DA synthesis are expressed by SH-SY5Y cells, including TH and VMAT2. Finally, SH-SY5Y cells express 45% of additional markers that are normally expressed by mature midbrain DAergic neurons including the transcription factor PITX3, the receptor tyrosine kinase RET and several neurotransmitter receptors (DRD2, and several subunits of GABA, NMDA, AMPA and ACh receptors). RA treatment does not change the expression of these mature SN DAergic markers, with the notable exception of RET, which is strongly induced. In contrast, RA treatment reduces expression of 2 out of 9 early stage DAergic markers. These data suggest that RA may shift the DAergic-like phenotype of SH-SY5Y cells towards a more mature state. Additionally, we observed an increase in DA immunoreactivity, suggesting that the net change of RA treatment is towards enhanced DA levels. This increase may in part be explained by the partial downregulation of the DA-degrading enzyme MAO-A, and the DA-converting enzyme DBH. Finally, our mRNA results suggest that both RA differentiated and non differentiated cells do not express detectible levels of the mRNA for the dopamine transporter (DAT) as the CT values in the qPCR were very low for this gene. On the other hand, we have shown that radioactive DA uptake is inhibited by the DAT specific blocker vanoxerine suggesting that this protein is expressed by the RA differentiated SH-SY5Y cells and is functional.

Furthermore, we observed a significant regulation of the DAergic receptor signaling pathway in the RA treated cells as indicated by IPA. The DA receptor signaling pathway involves DA binding to D1-type and D2-type DA receptors, and the activation of their respective downstream targets. Activation of the D1-type receptors promotes the activity of adenylate cyclases, and thus the conversion of ATP to cAMP. DA signaling via the D2-type receptors on the other hand inhibits the activity of adenylate cyclases. Our data indicate that signaling via the D2-type DA receptors is enhanced, as we observed a significant downregulation of adenylate cyclases. Furthermore, NCS1, an inhibitor of D2-type receptors, is also downregulated. These data suggest that the downstream targets of cAMP in this particular pathway (PKA and PP2A) should be decreased. In apparent contrast, we observed an increase in the expression of the B’beta subunits of protein phosphatase 2A (PPP2R2B and PPP2R5B). It is possible that PP2A subunit expression is enhanced via a DA receptor-independent mechanism. As the B’beta subunits dephosphorylate TH and thereby reduce its activity, we hypothesize that their upregulation may be part of a negative feedback loop to the DA synthesis pathway.

Together, our data show that RA treated cells, similarly to DAergic neurons *in vivo*, actively synthesize DA and are sensitive to DRD2-mediated DA receptor signaling and other neurotransmitter signaling pathways, including glutamate, acetylcholine and GABA. Even though SH-SY5Y cells share many characteristics with true DAergic cells, the observation that not all mature DA neurotransmitter-associated markers are expressed by SH-SY5Y cells does indicate, however, that SHSY-5Y do not fully phenocopy a true primary DAergic cell. Care should be taken when applying these cells in paradigms that require a full DAergic phenotype.

### RA Suppresses Serotonergic, Noradrenergic and Cholinergic Characteristics of SH-SY5Y Cells

In addition to investigating the DAergic phenotype of SH-SY5Y cells, the gene expression data also allowed us to assess the serotonergic, noradrenergic, cholinergic, glutaminergic, and histaminergic characteristics of RA-treated SH-SY5Y cells. The RA-induced downregulation of DBH and DDC, two key enzymes involved in noradrenaline and serotonin synthesis respectively, and the downregulation of noradrenaline-specifying transcription factor ASCL1 (fold change RA/noRA 0.27) and transcription regulator BMP7 (fold change RA/noRA 0.35) [60] all indicate that these phenotypes are at least partially suppressed in RA differentiated SH-SY5Y cells. Additionally, the observation that many of the monoamine producing enzymes and transporters are not expressed by the SH-SY5Y cells, irrespective of the treatment condition, suggests that these specific neurotransmitter phenotypes are not prominent in SH-SY5Y cells. However it should be noted that we cannot exclude that markers whose expression levels are below the microarray detection threshold are actually expressed at low levels.

### MPP(+) Treatment Induces Mitochondrial Stress in RA Differentiated SH-SY5Y Cells

A good cell model for PD related research should not only closely mimic the phenotype of SN DAergic neurons *in vivo*, but should also be sensitive to alterations in cellular processes that are commonly associated with PD. Mitochondrial dysfunction and increased oxidative stress are well known characteristics of PD [68] and MPP(+) has been widely used to induce PD-like mitochondrial impairments *in vitro* and *in viv*o [39]. We show here that RA differentiated SH-SY5Y cells 1) are capable of taking up MPP(+), most likely via dopamine (DAT) and noradrenalin (NAT) transporters, which mimics MPP(+) uptake by DAergic neurons *in vivo* and 2) are susceptible to mitochondrial dysfunction.

NoRA cells showed a larger decrease of ATP production compared to the RA differentiated cells. As indicated by our gene expression analysis, these cells continue to proliferate during the time in culture, requiring a high state of mitochondrial activity or higher numbers of active mitochondria. Therefore, the inhibiting effect of MPP(+) may induce larger effects on ATP production. We and others have observed that RA treatment of SH-SY5Y cells activates pro-survival mechanisms, such as Nrf2 or Akt signaling [21], which may underlie the differential sensitivity to MPP(+). Thus, measuring the effects of MPP(+) in RA-differentiated neuron-like cells may more accurately model mitochondrial dysfunction in DAergic neurons in the PD brain.

### Conclusion

Despite their neuron-like phenotype and expression of multiple DAergic markers, SH-SY5Y cells are neuroblastoma cells that only resemble mature midbrain SN DAergic neurons to some extent. However, SH-SY5Y cells can be cultured in a highly reproducible manner and allow for easy and successful genetic manipulation, including transfection-based gene knockdown or lenti-viral induced overexpression. Moreover, SH-SY5Y cells are readily available and can be easily expanded, and are thus suitable for use in high-throughput and high-content functional screening approaches to test large sets of target genes obtained by gene expression studies. Finally, the sensitivity of RA-treated SH-SY5Y to MPP(+) may be very useful in functional screening experiments investigating mitochondrial dysfunction in PD and the potential modifying effect of PD target genes towards the MPP(+) induced mitochondrial stress.

## Supporting Information

Figure S1 **The hybridization set up for RA vs. noRA microarray study.** Different culture days are indicated by numbers in the circle with each biological replicate (N = 3) being hybridized against a different culture time point.
(TIF)

Figure S2 **Retinoic acid receptor signaling pathway regulation in RA treated SH-SY5Y cells.** The figure represents the regulation of gene expression after RA treatment playing a role in retinoic acid signaling pathway illustrated by IPA. Red symbols represent genes significantly upregulated in RA treated SH-SY5Y cells and green represent genes significantly downregulated. Gray symbols with p values represent genes not significantly regulated after RA treatment.
(TIF)

Figure S3 **Expression profiles of all 20 gene clusters in time of culture in RA differentiated cells.** The expression level of each gene is indicated by the 2 log intensity (y axis) in time of culture (x axis). Each graph indicates the cluster number and the amount of genes present in this cluster. Red line indicates the average expression profile of each cluster.
(TIF)

Tables S1 Table S1. Gene validation by qPCR. Validation of microarray results with qPCR expression study of selected genes of interest. For the significantly regulated genes correlation coefficients are indicated between the pattern of array expression and qPCR expression. Sequences of qPCR detection primers forward and reverse are given. Table S2. The full list of significantly regulated genes showing expression differences between RA and noRA treated SH-SY5Y cells as indicated by the microarray analysis. Gene expression changes are given as fold changes between RA treated cells day 8 (D8) and day 1 (D1), between noRA- treated cells D8 and D1, and between RA and noRA treated cells on day 8. The cluster column indicates the cluster number to which the gene is allocated to (refer to Figure S1). Table S3. Ingenuity (IPA) and GO stat analysis of 4 clusters showing the most regulated expression of genes in the time of culture. The set of genes present in each indicated cluster was analyzed by two data bases and significant overrepresentations of gene functions are categorized with a color code: proliferation (red), cell death (blue), neuronal development (green), cellular development (gray) and development (yellow). Apart from the functional groups overrepresented in the specific clusters, p-values along with the number of molecules present in each group are shown in this table. Table S4. List of significantly regulated transcription factors during RA treatment of SH-SY5Y cells. Full list of all regulated transcription factors (TF’s) was composed with an algorithm after BH correction on the data set (see methods) (p value <0.05). Each TF was then investigated regarding its biological function with the relevant Entrez gene summary, GO annotation and IPA gene summary. The regulation pattern fold changes of each gene are indicated between day 1 (D1) and day 8 (D8) in RA and noRA culture conditions and between D8 of RA and noRA culture conditions. Additionally, a cluster number is shown to which each TF is assigned to based on its expression pattern (Figure S1).
(XLSX)
