# Supplementary figures and images for: Phenotypic Characterization of Retinoic Acid Differentiated SH-SY5Y Cells by Transcriptional Profiling
Source: PLoS One. 2013 May 28;8(5):e63862. doi: 10.1371/journal.pone.0063862 (PMC3665836; doi:10.1371/journal.pone.0063862)

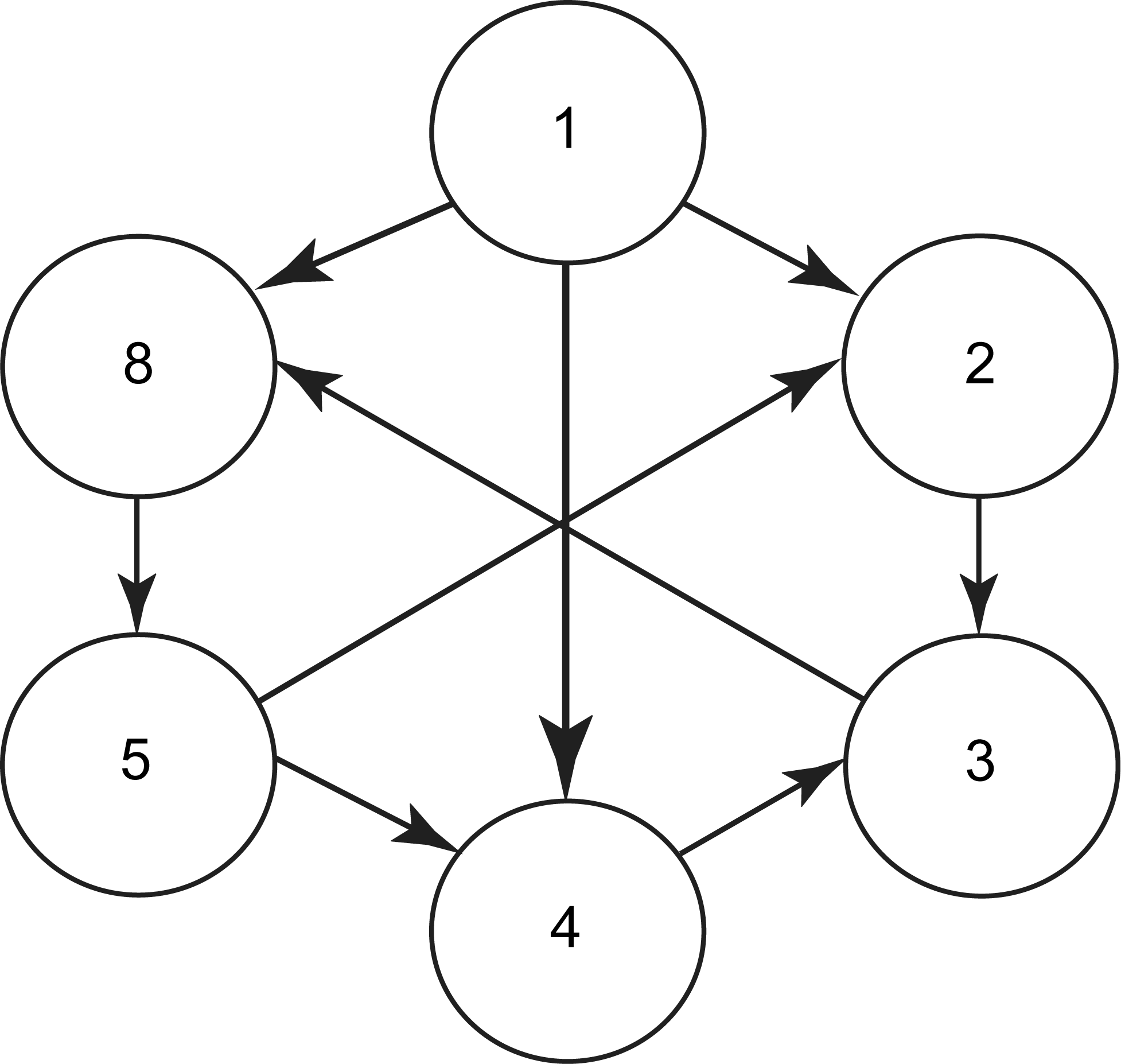

Supplement: Figure S1 — The hybridization set up for RA vs. noRA microarray study. Different culture days are indicated by numbers in the circle with each biological replicate (N = 3) being hybridized against a different culture time point. (TIF) [file pone.0063862.s001.tif]

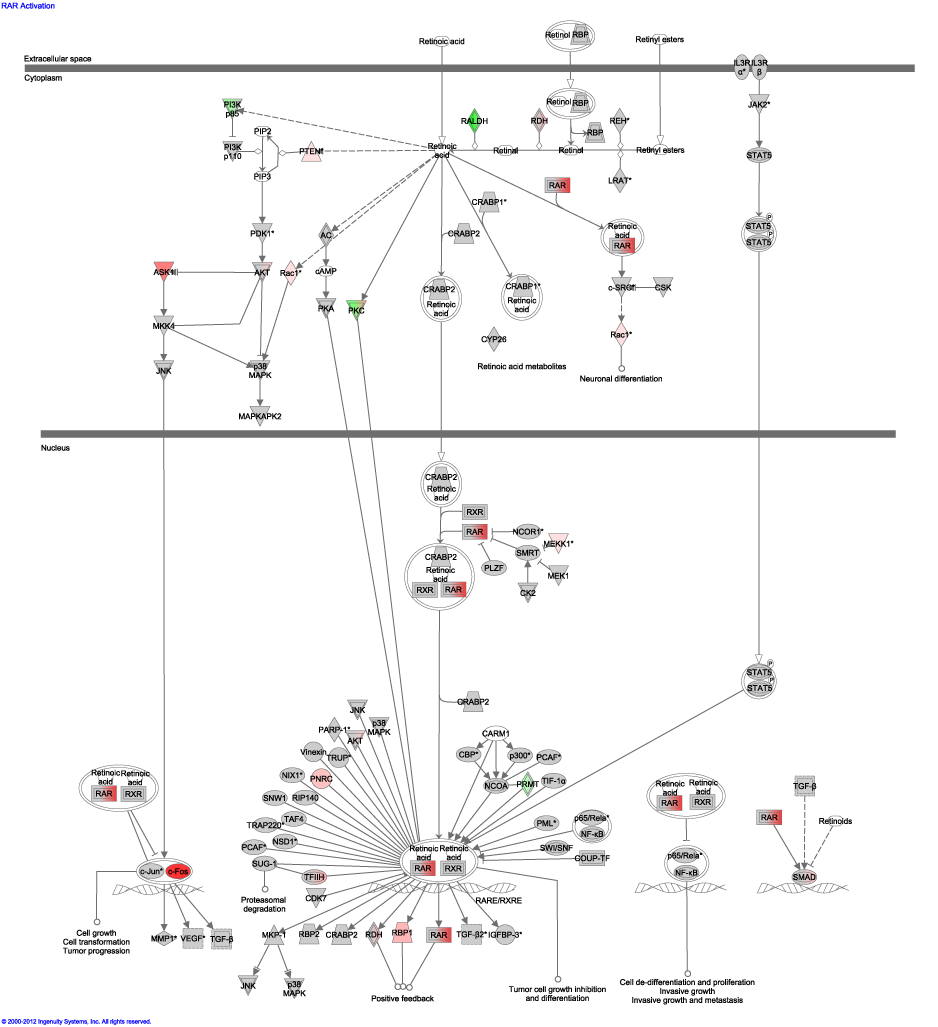

Supplement: Figure S2 — Retinoic acid receptor signaling pathway regulation in RA treated SH-SY5Y cells. The figure represents the regulation of gene expression after RA treatment playing a role in retinoic acid signaling pathway illustrated by IPA. Red symbols represent genes significantly upregulated in RA treated SH-SY5Y cells and green represent genes significantly downregulated. Gray symbols with p values represent genes not significantly regulated after RA treatment. (TIF) [file pone.0063862.s002.tif]

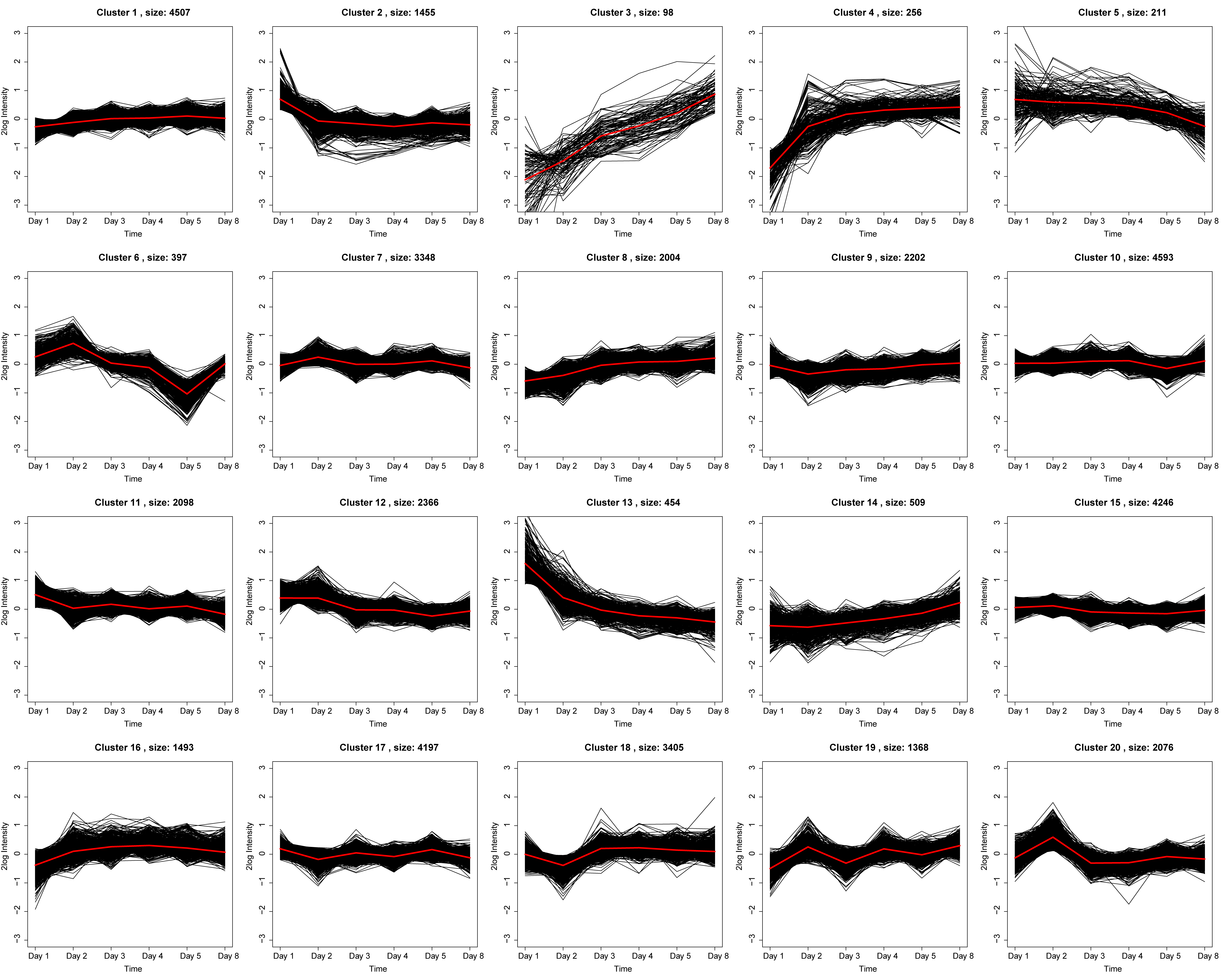

Supplement: Figure S3 — Expression profiles of all 20 gene clusters in time of culture in RA differentiated cells. The expression level of each gene is indicated by the 2 log intensity (y axis) in time of culture (x axis). Each graph indicates the cluster number and the amount of genes present in this cluster. Red line indicates the average expression profile of each cluster. (TIF) [file pone.0063862.s003.tif]
